# Sacrificing Alginate in Decellularized Extracellular Matrix Scaffolds for Implantable Artificial Livers

**DOI:** 10.3390/jfb16010035

**Published:** 2025-01-19

**Authors:** Chanh-Trung Nguyen, Van Phu Le, Thi Huong Le, Jeong Sook Kim, Sung Hoon Back, Kyo-in Koo

**Affiliations:** 1 Department of Electrical, Electronic and Computer Engineering, University of Ulsan, Ulsan 44610, Republic of Korea; nctrung1407@mail.ulsan.ac.kr (C.-T.N.);; 2 Department of Obstetrics and Gynecology, Ulsan University Hospital, University of Ulsan College of Medicine, Ulsan 44033, Republic of Korea; jeongsookkim@uuh.ulsan.kr; 3 School of Biological Sciences, University of Ulsan, Ulsan 44610, Republic of Korea; 4 Basic-Clinical Convergence Research Institute, University of Ulsan, Ulsan 44610, Republic of Korea

**Keywords:** decellularized extracellular matrix, implantation, liver tissue, alginate, alginate lyase

## Abstract

This research introduced a strategy to fabricate sub-millimeter-diameter artificial liver tissue by extruding a combination of a liver decellularized extracellular matrix (dECM), alginate, endothelial cells, and hepatocytes. Vascularization remains a critical challenge in liver tissue engineering, as replicating the liver’s intricate vascular network is essential for sustaining cellular function and viability. Seven scaffold groups were evaluated, incorporating different cell compositions, scaffold materials, and structural configurations. The hepatocyte and endothelial cell scaffold treated with alginate lyase demonstrated the highest diffusion rate, along with enhanced albumin secretion (2.8 µg/mL) and urea synthesis (220 µg/mL) during the same period by day 10. A dense and interconnected endothelial cell network was observed as early as day 4 in the lyased coculture group. Furthermore, three-week implantation studies in rats showed a stable integration to the host with no adverse effects. This approach offers significant potential for advancing functional liver tissue replacements, combining accelerated diffusion, enhanced albumin secretion, improved urea synthesis, dense vascular network formation, and stable implantation outcomes.

## 1. Introduction

Tissue engineering focuses on creating functional tissues to replace or repair damaged or diseased ones [1]. A critical challenge in this field is achieving vascularization, the formation of blood vessels within engineered tissues, which is essential for delivering nutrients, oxygen, and removing waste [2,3]. Without an adequate vascular network, large tissue constructs often fail due to necrosis and metabolic insufficiency [4].

The liver, as a highly vascularized organ, relies on a complex network of blood vessels for optimal function [5]. This vasculature supports oxygen and nutrient delivery to hepatocytes while removing waste and toxins [6]. Replicating the liver’s intricate vascular architecture in engineered tissues remains a significant challenge [7,8]. Approaches to enhance vascularization include angiogenic growth factors, pre-vascularized scaffolds, and coculturing endothelial cells with other cell types [9,10].

Biomaterials play a crucial role in supporting cell growth and function in liver tissue engineering. While alginate hydrogels are valued for their biocompatibility and ease of gelation, they lack bioactive sites for cell adhesion, restricting cell attachment, migration, and spreading [11,12,13]. In contrast, the liver decellularized extracellular matrix (dECM) provides a biologically relevant environment, rich in collagen and adhesion sites, enhancing cellular interactions and mimicking natural liver tissue [14]. Combining alginate and the liver dECM offers a hybrid scaffold that merges the biological advantages of the dECM with alginate’s structural stability [15,16,17].

Various methods exist for engineering liver tissue, including micropatterned coculture, spheroid formation, bioprinting, and decellularization–recellularization [18,19,20,21,22,23,24,25,26]. Among these, bioprinting stands out for its precision in organizing cells and biomaterials in 3D, overcoming limitations of other techniques such as a low spatial resolution and limited design flexibility.

Recent studies highlight the ongoing challenges in vascularization. For instance, Laschke et al. demonstrated limited angiogenesis in polyurethane scaffolds, even after incorporating nanosized hydroxyapatite particles [27,28]. Similarly, Kurashina et al. observed insufficient vascularization in microfibers cocultured with HepG2 and human umbilical vein endothelial cells, emphasizing the need for improved biomaterials and techniques [29].

**Figure 1:**
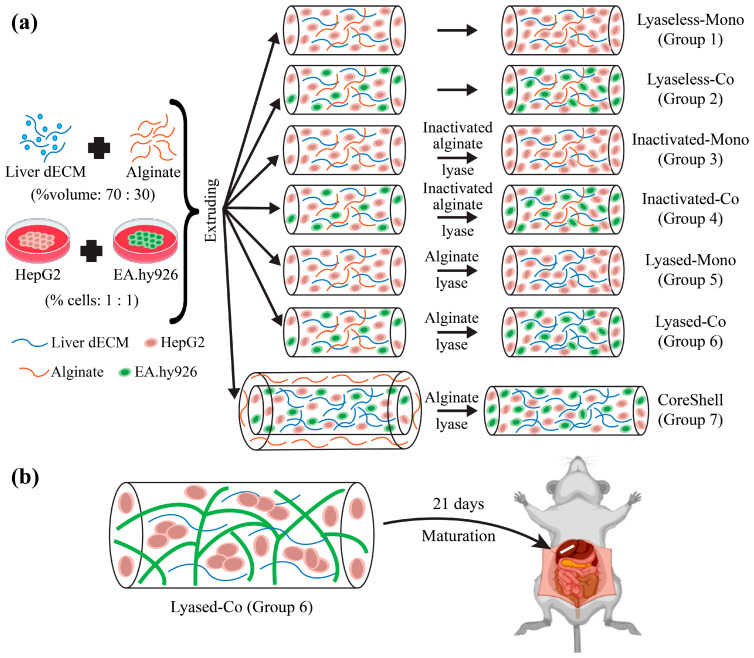
The schematic diagram of this study. (**a**) The fabrication condition of the seven groups. (**b**) The implantation experiment of the lyased coculture scaffold (Group 6).

In this study, we explored the optimization of liver scaffolds using various cell types, scaffold materials, and structures. Seven fibrous scaffolds were extruded, as shown in Figure 1. Hepatic cells (HepG2) and endothelial cells (EA.hy926) together were encapsulated in Groups 2, 4, 6, and 7 to compare with Groups 1, 3, and 5 having hepatic cells alone. A mixture of alginate and the liver dECM was extruded and 24 h later some groups (Groups 5 and 6) were treated with alginate lyase to dissolve the alginate, which were named as the lyased groups. For comparison, other groups (Groups 1 and 2) were matured without any alginate lyase treatment, so they were called the lyaseless groups. Groups 3 and 4 were applied with inactivated alginate lyase treatment, so they were called the inactivated groups. Meanwhile, Group 7 had a coaxial two-layer structure: the core layer encapsulated hepatic cells and endothelial cells together in the liver dECM and the outer layer comprised alginate alone without any cell. The alginate outer layer was dissolved 24 h after the extrusion. All the groups were characterized by their scaffold diameter, cell viability, albumin secretion, urea synthesis, and cell morphologies. The lyased coculture scaffold (Group 6) was implanted in a rat to evaluate its implantability.

## 2. Materials and Methods

### 2.1. Hydrogel

a. Liver dECM synthesis

The porcine liver tissue was sliced into 1 mm-thick sections and thoroughly rinsed twice with cold PBS for an hour at 500 rpm. Three detergent solutions were prepared to treat the sliced liver tissue. First, sodium dodecyl sulfate (SDS, Sigma-Aldrich, St. Louis, MO, USA) was diluted in deionized (DI) water to create concentrations of 0.1%, 1%, and 2%. Second, 1% Triton X-100 (Sigma-Aldrich, St. Louis, MO, USA) and a combination of 1% SDS and 1% Triton X-100 were diluted in DI water to form the second and third treatment solutions. Next, the tissue sections were immersed in each chemical solution for 48 h at room temperature with constant agitation at 500 rpm, replacing the detergent every 8 h. After decellularization, the tissue was flushed three times with PBS for 2 h each to eliminate residual detergent and cellular debris. The samples were sterilized using 100% and 70% ethyl alcohol for 30 min and 3 h, respectively. Lastly, the sterilized samples were rinsed thrice with PBS containing 1% penicillin/streptomycin for 2 h each.

The decellularized samples were freeze-dried for 72 h and ground into powder using a blender. Three hundred fifty milligrams of the liver decellularized extracellular matrix was solubilized with 100 mg of pepsin (Sigma-Aldrich, St. Louis, MO, USA) in 100 mL of 5% acetic acid (Sigma-Aldrich, St. Louis, MO, USA). The mixture was stirred at room temperature for four days. After complete digestion, the dECM solution was acquired by centrifuging at 3000 rpm for 15 min to remove particles and then stored at 4 °C for future use.

b. Mixture of liver dECM and alginate

A 35 mg/mL solution of the dECM was prepared by diluting it in 10X PBS (Sigma-Aldrich, St. Louis, MO, USA) and neutralizing it with 10 M and 1 M sodium hydroxide (NaOH, VWR International, Radnor, PA, USA). Deionized water was added to achieve a 30 mg/mL liver dECM concentration. This process was conducted on ice to prevent dECM gelation. Sodium alginate powder (Daejung Chemicals, Siheung, Republic of Korea) was dissolved in DI water to create 1.5% (*w*/*v*) solution, which was then sterilized at 110 °C for 30 min before biological experiments. A bioink was formulated by combining the liver dECM and sodium alginate at a 70:30 volume ratio and stored at 4 °C before constructing the scaffolds.

### 2.2. Cell Culture

HepG2 and EA.hy926 cell lines were purchased from the American Type Culture Collection (ATCC, Manassas, VA, USA) and cultured in DMEM High Glucose (Dulbecco’s Modified Eagle Medium, Welgene, Gyeongsan, Republic of Korea) supplemented with 10% fetal bovine serum (Gibco, Thermo Scientific, Waltham, MA, USA). The fresh culture mediums were changed every 2–3 days. They were cultured in a humidified incubator at 37 °C with 5% CO_2_ and were passaged before reaching approximately 80% surface coverage.

### 2.3. Scaffold Generation

To demonstrate the long-term functionality of the engineered liver tissue, we proposed seven groups with variations in their structures, materials, and culture conditions. These are categorized into seven groups and listed in Table 1. Hepatic cells (HepG2) only were encapsulated by a mixture of the dECM and alginate (Group 1). Its mixture ratio was seventy-to-thirty. The Group 2 scaffold contained hepatic cells and endothelial cells (EA.hy926) together with the hydrogel mixture. The cell ratio was one-to-one. Group 3 contained only hepatic cells encapsulated in a mixture of the dECM and alginate, treated with inactivated alginate lyase 24 h after extrusion. Group 4 consisted of both hepatic cells and endothelial cells encapsulated in the same hydrogel mixture, also treated with inactivated alginate lyase, with a one-to-one cell ratio. In the case of Group 5, the hepatic cell scaffold was treated with alginate lyase 24 h after its extrusion. Group 6 lyased alginate in the scaffold including hepatic cells and endothelial cells. The outer layer of Group 7 comprised alginate and its inner layer had hepatic cells and endothelial cells together in the dECM. The outer alginate of Group 7 was dissolved.

**Figure 2:**
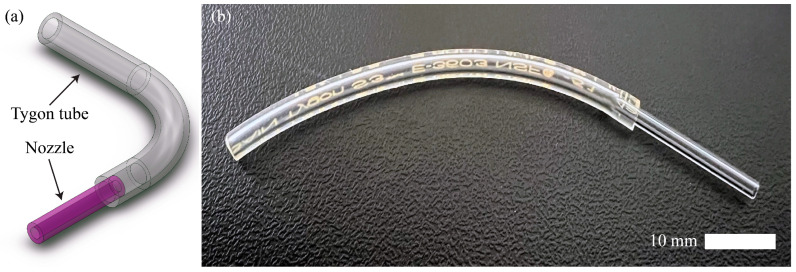
The monolithic device for extruding the monolithic-layer scaffolds (Groups 1, 2, 3, 4, 5, and 6). (**a**) The schematic diagram of the device. (**b**) The monolithic device.

For the monolithic-layer scaffolds (Groups 1, 2, 3, 4, 5, and 6), a simple device comprising one glass capillary (1160 μm internal diameter (ID), G200-3, Warner Instruments LLC, Holliston, MA, USA) and a Tygon tube (Saint-Gobain, Courbevoie, France) (Figure 2) was utilized. The 1160 μm-diameter glass capillary was connected through the Tygon tube to a syringe pump for the controlled injection of bioink. The bioink consisted of the desired material mixed with 10^7^ cells (monoculture: HepG2 only, coculture: HepG2 and EA.hy926). The bioink was injected at a flow rate of 0.5 mL/min. The extruded monolithic-layer scaffold was submerged into a 0.1 M CaCl_2_ (Daejung Chemicals, Siheung, Republic of Korea) bath to cross-link.

**Figure 3:**
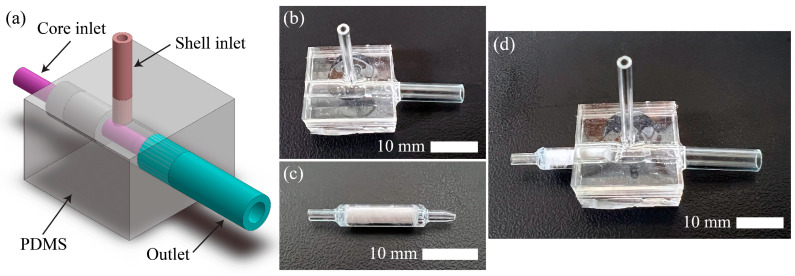
The coaxial device for extruding the CoreShell scaffold (Group 7). (**a**) The schematic diagram of the device. (**b**) The PMDS body with the shell inlet and the outlet. (**c**) The core inlet. (**d**) The completed CoreShell device.

For the CoreShell scaffold (Group 7), another device was fabricated, as shown in Figure 3. It consisted of three glass capillaries: one 1160 μm-ID tube for the inner-core inlet, another 1160 μm-ID tube for the outer-shell inlet, and one 2.4 mm-diameter glass tube for the outlet (1653155, Bio-rad, Hercules, CA, USA). All the tubes linked each other in a block of polydimethylsiloxane (PDMS, Dow Corning Corporation, Midland, MI, USA). The fabricated devices were sterilized at 121 °C for 15 min before biological experiments.

To generate the CoreShell scaffolds, two syringe pumps (11 Elite C300918, Harvard Apparatus, Holliston, MA, USA) were connected to the CoreShell device through Tygon tubes. For the core inlet, a mixture of 30 mg/mL liver dECM, 10^7^ cells/mL HepG2-EA.hy926, and 0.1 M CaCl_2_ was supplied. For the shell inlet, 1.5% *w*/*v* sodium alginate was supplied to the shell of the scaffold. The extruded scaffold was submerged into a 0.1 M CaCl_2_ bath through the outlet and cross-linked. All groups were cultured in an incubator at 37 °C with 5% CO_2_ and replaced with a fresh medium every day.

Alginate lyase solution (0.05 units/mL, Sigma-Aldrich, St. Louis, MO, USA) was heat-denatured at 105 °C for 15 min to produce the inactivated alginate lyase [30]. After 24 h of culturing, Groups 3 and 4 were treated with the inactivated alginate lyase. Additionally, Groups 5, 6, and 7 were treated with 0.05 units/mL active alginate lyase to remove the alginate component from the scaffolds.

### 2.4. Diffusion Assay

To analyze the diffusibility of every group, the cultured scaffolds were submerged in 25 mg/mL fluorescent dextran solution (Sigma-Aldrich, St. Louis, MO, USA) on day 10. The soaked scaffolds were taken out of the solution and subsequently put into an empty dish. Under an IX53 inverted fluorescent microscope (Olympus, Tokyo, Japan), the fluorescence diffusion from the scaffolds was analyzed quantitatively after waiting five minutes for the fluorescence solution to flow out of the scaffolds.

### 2.5. Cell Viability

The viability and proliferation of the cultured scaffolds were evaluated at days 1, 4, 7, and 10 using live/dead viability kits for mammalian cells (L3224, Thermo Scientific, Waltham, MA, USA). The scaffold’s concentration was 0.05% of Calcein AM (4 mM) in anhydrous dimethyl sulfoxide (DMSO) and 0.2% ethidium homodimer-1 (2 mM) in DMSO/H_2_O at 1:4 (*v*/*v*). A stained scaffold was washed 3 times in PBS for 5 min and then observed under a fluorescent microscope. The fluorescent intensity of live cells (green channel) and dead cells (red channel) was analyzed by ImageJ software (version 1.54k, Fiji, NIH Image, Bethesda, MD, USA). The percentage of cell viability was calculated using the ratio between the green intensity and the summation of the green and red intensity.

### 2.6. Cytotoxicity Assay

A colorimetric method was used to assess cytotoxicity in seven groups on days 1, 4, 7, and 10. Scaffolds measuring 2 mm in length were placed in a 96-well plate with 100 μL of medium per well. Subsequently, 20 μL of the CellTiter 96^®^ AQueous One Solution Cell Proliferation Assay (Promega, Madison, WI, USA), containing a tetrazolium compound (3-[4,5-dimethylthiazol-2-yl]-5-[3-carboxymethoxyphenyl]-2-[4-sulfophenyl]-2H-tetrazolium; MTS) and an electron coupling reagent (phenazine ethosulfate; PES), was added. The scaffolds were then incubated at 37 °C for 4 h. Absorbance was measured at 490 nm using a SpectraMax iD3 reader (Molecular Devices, San Jose, CA, USA).

### 2.7. Measurement of Scaffold Mechanical Properties via Tensile Strength

**Figure 4:**
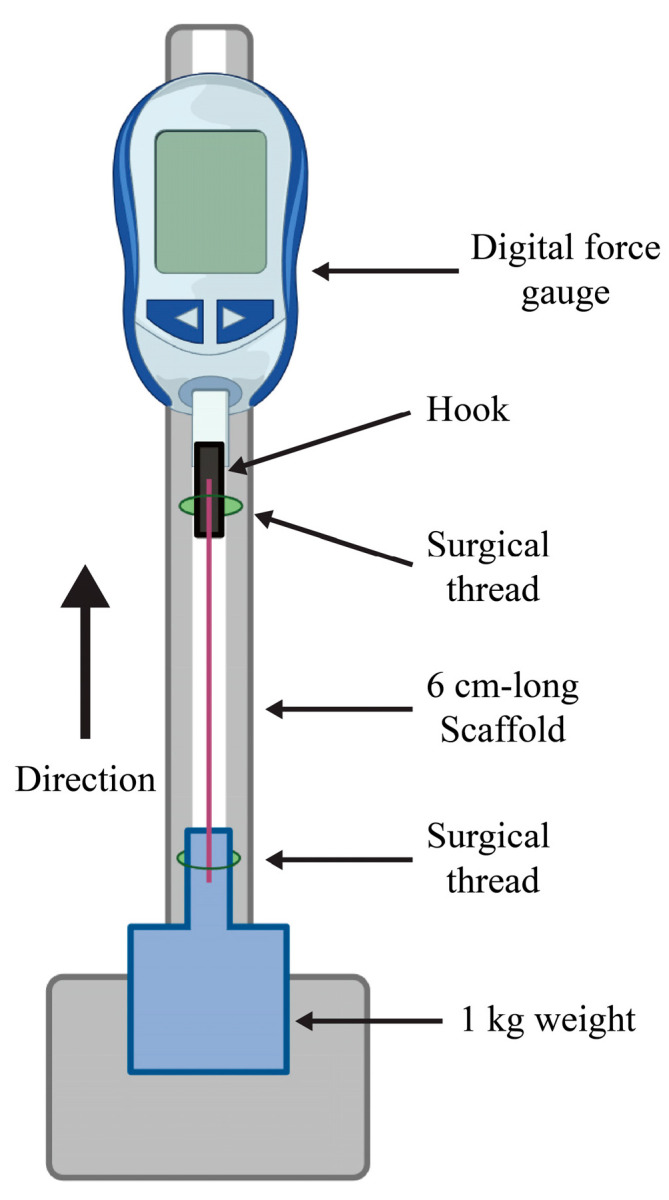
The schematic diagram of the tensile strength system.

A custom-built system was employed to measure the tensile strength of matured scaffolds from the seven groups, as illustrated in Figure 4. A 6 cm-long scaffold cultured for 10 days was prepared for testing. One end of the scaffold was securely attached to a hook accessory on a digital force gauge (DSV-2N, Imada, Japan) using a surgical thread to ensure a firm connection. Similarly, the other end of the scaffold was tied to a 1 kg weight using the surgical thread.

The tensile test was performed in a bottom-to-top direction. The force gauge was gradually raised, applying tension to the scaffold until failure occurred. At the breaking point, the maximum tensile force was recorded using the digital force gauge. The tensile strength was calculated by dividing the maximum tensile force at failure (in Newtons) by the cross-sectional area of the scaffold (in square meters).

### 2.8. Liver Function

For albumin and urea secretion, the total amount of albumin and urea released from the cultured scaffolds into the cell culture medium for 24 h was analyzed on day 1, 4, 7, and 10 by an enzyme-linked immunosorbent assay (ELISA kit, Abcam, Cambridge, UK) and the urea assay kit (Abcam, Cambridge, UK) according to the manufacturer’s instructions, respectively. Moreover, in the animal experiment albumin and urea concentrations from blood serum samples were also measured using the same kits on day 7, 14, and 21, and one day prior to surgery.

### 2.9. Immunofluorescence Staining

To observe albumin secretion, cell–cell adhesion, and cell nuclei, the cultured scaffolds were stained using anti-albumin, anti-CD31, and DAPI, respectively. Initially, they were fixed with 3.5% paraformaldehyde (PFA, P6148, Sigma-Aldrich, St. Louis, MO, USA) for 40 min at room temperature. The fixed scaffold was then immersed in 3% gelatin solution and incubated at 37 °C for gelation. Subsequently, the scaffolds in gelatin were permeabilized with 0.5% Triton X-100 (Sigma-Aldrich, St. Louis, MO, USA) for 5 min at room temperature. A mixture of primary antibodies, including anti-albumin (Abcam, Cambridge, UK) and anti-CD31 (Abcam, Cambridge, UK), was incubated overnight at 4 °C. Afterward, secondary antibodies (Abcam, Cambridge, UK) were applied for 2 h at room temperature. Cell nuclei were stained with DAPI (D1396, Invitrogen, Waltham, MA, USA) for 5 min. Following each chemical treatment, the scaffolds were washed 3 times with PBS for 5 min. The stained samples were observed using a fluorescent microscope and an FV1000 laser scanning confocal microscope (Olympus, Tokyo, Japan).

### 2.10. In Vivo Experiment

Sprague Dawley male rats (5 weeks old, 95–135 g) were purchased from Hana company (Busan, Republic of Korea). The rats were kept in a facility with 60% humidity at 24 °C, a 12 h light/dark cycle, and free access to drinks and food. Animal studies were performed per the principles and guidelines of laboratory animal care and ethics, with permission from the University of Ulsan’s Institutional Animal Care and Use Committee (GIG-22-010, University of Ulsan, Ulsan, Republic of Korea). The experiment was conducted three times, using a total of nine rats.

A rat was placed in an individual box without feeding for 1 day in a noise-free area before surgery. All surgical instruments were sterilized by an autoclave at 121 °C for 30 min. Rats were divided into three groups: sham, hepatectomy, and implantation. Three groups were followed on day 7, 14, and 21. They were anesthetized using 20 mg/mL 2,2,2-Tribromoethanol (Sigma-Aldrich, St. Louis, MO, USA). The sedative period typically lasts for 60 min. Their hair was removed by Nair gel (Church & Dwight, UK), and povidone-iodine 10% (Firson, Cheonan, Republic of Korea) was applied for disinfection before surgery. The skin on the abdomen was incised appropriately 5 cm in length and 3 cm in width from the cartilaginous section at the lower end of sternum with scissors and forceps. An eye speculum was put in the opened area to observe the abdominal cavity. A dissection forceps was used to extract the left liver lobe and place it on a glass slide. A sterile scalpel blade (#15) was used to perform a hepatectomy (1 × 3 mm). A swab was used to maintain the cutting liver area for 5 min to prevent the liver from bleeding.

The right liver lobe was opened in the implantation groups, and a 3D-engineered liver scaffold (1 × 3 mm) was inserted into this site. Then, the connection between the opened liver lobe and the liver scaffold was sutured using 7-0 silk sutures (Ailee, Busan, Republic of Korea) to avoid the displacement of the scaffold. After that, the liver was returned to the abdominal cavity, and the abdominal wall and skin were sutured with 3-0 nylon sutures (Ailee, Busan, Republic of Korea). The surgical incision was cleaned with a surgical iodine-soaked swab twice. The surgical rats were placed in an individual box in a noise-free area. The implanted rats were observed in the recovery period and their food and water consumption was monitored for 3 h.

Blood samples were collected from the lateral tail veins of the rats one day before surgery, and on days 7, 14, and 21. These samples were centrifuged at 2000× *g* for 10 min at room temperature to gather the serum. The liver function tests (albumin and urea) were performed using the collected serum with an ELISA and urea assay kit. The surgical rats were anesthetized again on days 7, 14, and 21 to harvest the implanted liver region. After harvesting, cervical dislocation was performed on the anesthetized rats until respiration stopped for euthanasia.

### 2.11. Histological Analysis

The cell scaffolds were fixed overnight in 3.5% PFA before dehydrating in ethanol. Before wax infiltration, xylene was used to replace the ethanol in the samples. Then, the samples were sliced into thicknesses of 10 μm using RM2255 microtome (Leica Biosystems, Nussloch, Germany). Before staining, the paraffin wax in the sections was removed using xylene before rehydrating in ethanol and water. These processes were performed at the Bio-Medical Institute at Kyoungpook National University Hospital, Dae-gu, Republic of Korea. For H&E staining, the slices were stained with Harris Haematoxylin (3801561, Leica Biosystems, Nussloch, Germany) and Alcoholic Eosin Y515 (3801610, Leica Biosystems, Nussloch, Germany). Then, they were treated with ammonia buffer (50 mL deionized water + 0.15 mL ammonia solution (NH_4_OH)). The H&E-stained slices were observed under a bright-field microscope (IX71, Olympus, Tokyo, Japan).

### 2.12. Statistical Analysis

The results were represented with a mean value ± one standard error from at least three independent repetitions. One-way ANOVA and Tukey’s post hoc test were utilized to evaluate the statistical significance level. Its significance is remarked as * for *p* < 0.05, ** for *p* < 0.01, and *** for *p* < 0.001.

## 3. Results and Discussion

### 3.1. Measurement of Scaffold Diameter

**Figure 5:**
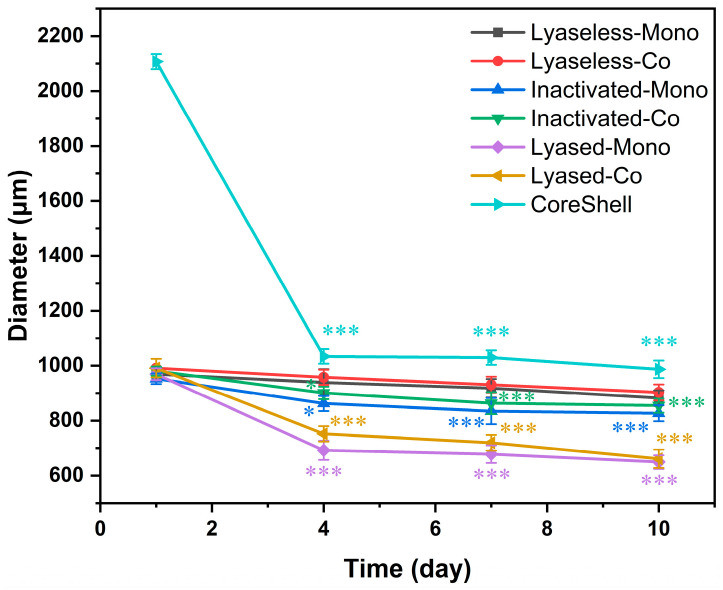
The reductions in scaffold diameter for 10 days with the seven different groups (* for *p* < 0.05, *** for *p* < 0.001, and *n* = 6).

The diameter changes in the cultured scaffolds from day 1 to day 10 highlighted the influence of scaffold composition, structure, and enzymatic treatment on their physical stability, as shown in Figure 5. On day 1, all the groups, except the CoreShell group (Group 7), started with a similar diameter of approximately 1000 µm, reflecting consistent initial extrusion and structural uniformity. In contrast, the CoreShell group had a significantly larger diameter of about 2100 µm due to its outer alginate layer.

All the groups exhibited a decrease in diameter over time. The three lyase-treated groups (Groups 5, 6, and 7) exhibited significant shrinkage after the alginate removal by lyase treatment on day 1, and it was particularly noticeable by day 4. The CoreShell scaffold (Group 7) displayed the most substantial decrease, stabilizing at 1030 µm, which highlights the role of the alginate shell in providing initial thickness. Meanwhile, the lyased groups (Groups 5 and 6) continued to shrink, reaching approximately 650 µm by day 10. This pronounced compaction is attributed to their uniform composition and dependence on the dECM and cells for structural integrity post-alginate degradation.

The inactivated lyased groups (Groups 3 and 4) showed intermediate shrinkage between the lyased and lyaseless groups. Group 3 (Inactivated-Mono) displayed a gradual reduction in diameter, stabilizing at around 830 µm by day 10. The inactivated enzyme’s limited breakdown of alginate could slightly enhance structural retention compared to the lyase-treated groups, preserving some initial scaffold integrity. However, the absence of coculture in Group 3 may have restricted further stabilization through cell–cell interactions. Group 4 (Inactivated-Co) exhibited a better stability than Group 3, stabilizing at approximately 850 µm by day 10. The coculture condition in Group 4 likely enhanced cell–matrix interactions and contributed to a more compact and cohesive structure, minimizing shrinkage.

In contrast, the lyaseless groups (Groups 1 and 2) showed a more gradual reduction in diameter, decreasing only slightly to around 900 µm by day 10. The absence of alginate degradation in these groups contributed to greater structural retention, with the minor reduction likely caused by cellular proliferation and matrix remodeling. Notably, the Lyaseless-Co group (Group 2), containing both hepatocytes and endothelial cells, may have experienced subtle remodeling due to cell–cell interactions and matrix contraction.

### 3.2. Diffusion Profile

**Figure 6:**
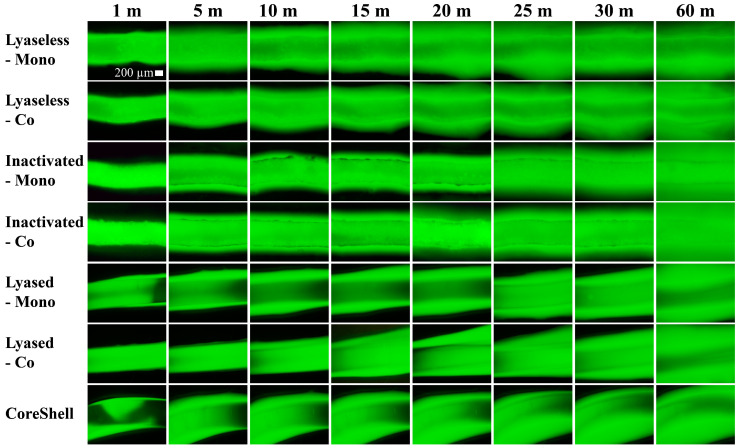
The time-lapse images of diffusion scaffolds up to 60 min from the seven groups. (Scale bar: 200 µm).

To evaluate diffusion, a dextran solution was introduced into all seven scaffolds for 60 min. A series of the fluorescence microscope images (Figure 6) illustrates dextran diffusion as time progressed.

**Figure 7:**
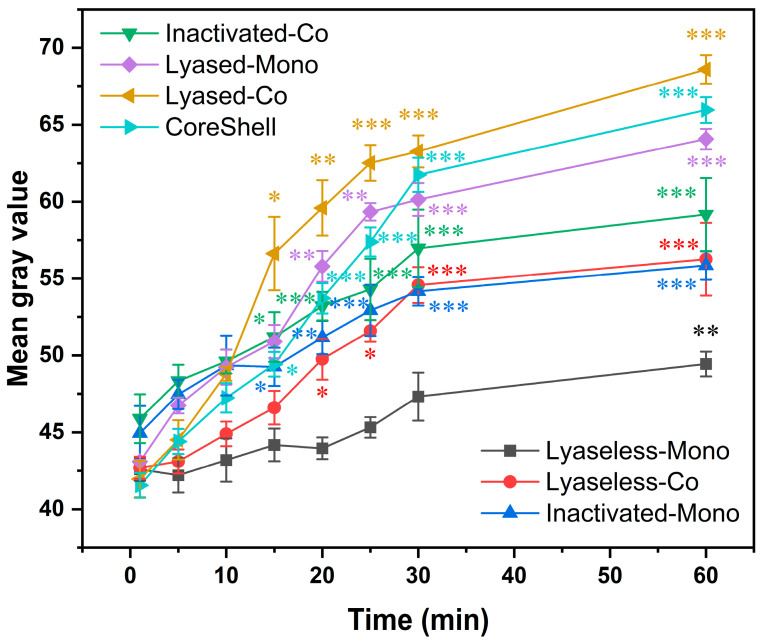
The analysis of mean gray values was conducted on the time-lapse images of all the seven scaffolds across the time range of 1-to-60 min (* for *p* < 0.05, ** for *p* < 0.01, *** for *p* < 0.001, and *n* = 4).

Quantitative analysis of the mean gray values from the fluorescence images (Figure 7) provided deeper insights into these diffusion patterns observed in the scaffolds. During the first 10 min, no significant differences in mean gray values were observed among the groups, suggesting a uniform rate of diffusion initially. Over 10 min, notable distinctions in diffusion performance appeared.

The Lyaseless-Mono (Group 1) and Inactivated-Mono (Group 3) scaffolds exhibited a lower diffusion efficiency compared to the other groups. The diffusion in Group 1 was slow and limited, indicating that the dense alginate matrix restricted molecular transport. In contrast, Group 3 showed slightly improved diffusion, suggesting that the partial breakdown of alginate, even when treated with inactivated alginate lyase, enhanced permeability to a minor extent.

The inactivated lyase groups (Groups 3 and 4) displayed an intermediate diffusion trend between the lyase-treated and lyaseless scaffolds. Group 3 (inactivated alginate lyase—monoculture) showed a gradual increase in mean gray values, reflecting a moderate diffusion capacity. However, its diffusion efficiency was lower than that of the inactivated coculture group (Group 4) at all the measured time points. This difference highlights the role of cell distribution in enhancing molecular transport, with the coculture condition likely forming a more interconnected cell network that facilitates diffusion.

In comparison, the lyase-treated groups (Groups 5, 6, and 7) exhibited significantly higher mean gray values than the lyaseless and inactivated groups (Groups 1, 2, 3, and 4). Among these, the lyased coculture scaffold (Group 6) showed the best diffusion performance. By 60 min, Group 6 achieved the highest mean gray value, emphasizing the combined effects of coculture and lyase treatment. Meanwhile, the CoreShell scaffold (Group 7) exhibited slightly lower mean gray values compared to Group 6. This difference may be attributed to its larger diameter, which could impede prolonged diffusion to some extent. However, the difference was not statistically significant.

In summary, while the inactivated groups (Groups 3 and 4) displayed improved diffusion characteristics compared to the lyaseless group (Groups 1 and 2), they fell short of the diffusion efficiency observed in the lyase-treated groups. These results demonstrated that lyase treatment significantly enhances scaffold permeability, particularly in the coculture condition (Group 6), which achieved the highest diffusion efficiency. The CoreShell scaffold (Group 7) also exhibited strong diffusion properties, although these were slightly lower than Group 6. These findings underscore the importance of scaffold composition, culture conditions, and structural design in optimizing diffusion characteristics, which are critical for efficient nutrient and oxygen transport in engineered liver tissues.

### 3.3. Cell Viability

**Figure 8:**
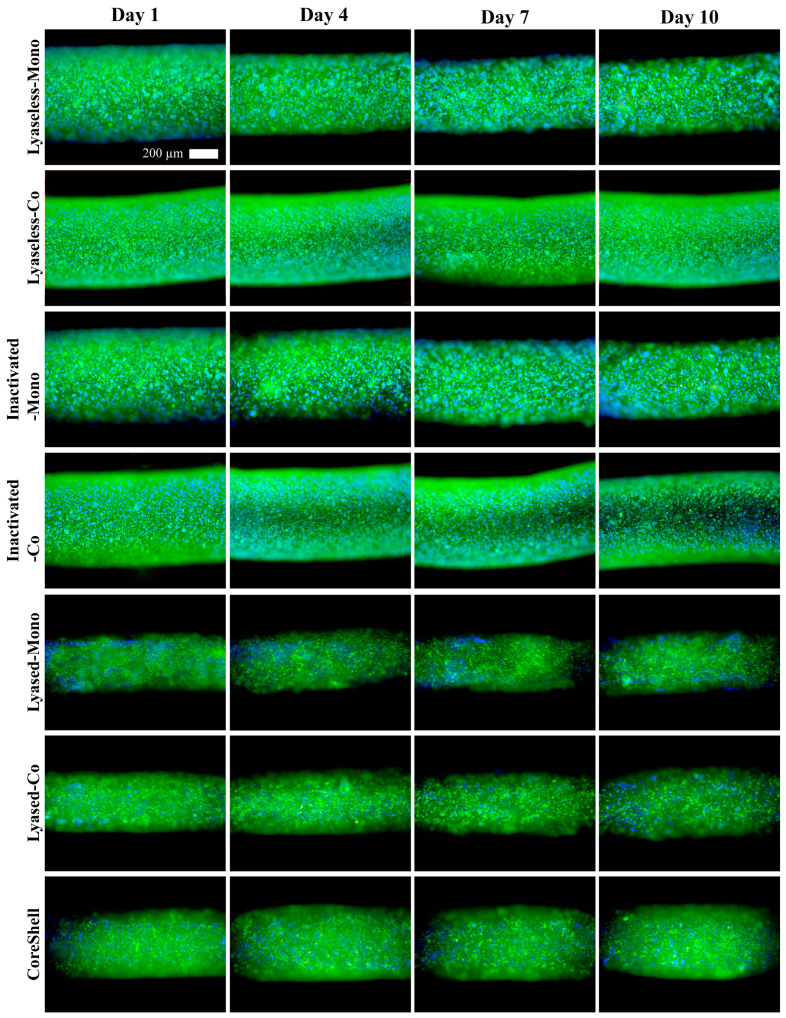
The live/dead-stained scaffolds up to day 10 in the seven groups (green: live cells, red: dead cells, blue: nuclei, scale bar: 200 μm) (*n* = 4).

**Figure 9:**
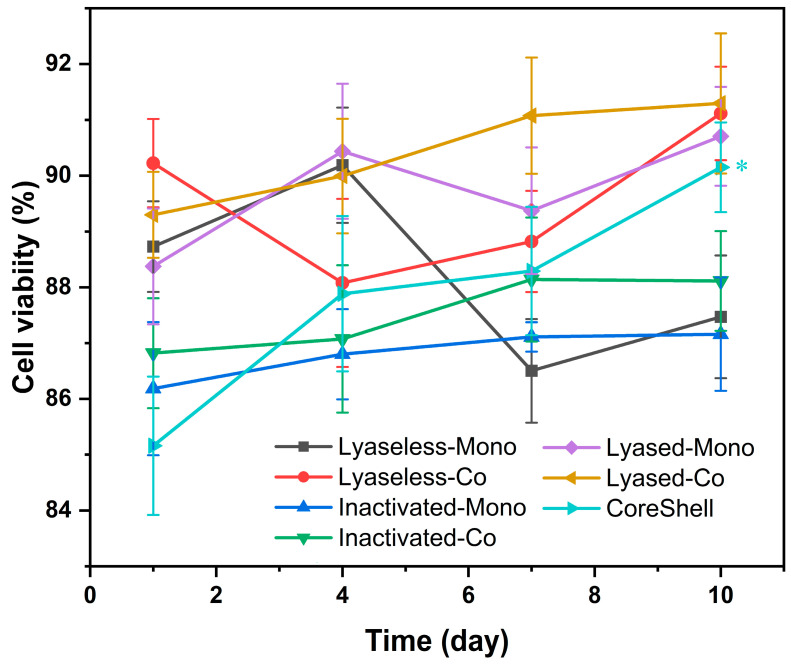
The cell viability of all seven scaffolds is shown up to 10 days (* for *p* < 0.05, and *n* = 4).

The cell viability of cultured scaffolds, as visualized in Figure 8 and quantified in Figure 9, reveals key trends in cell health and proliferation across various scaffold groups according to time. Fluorescence microscopy images (Figure 8) show cell nuclei as blue, live cells as green, and dead cells as red. Although the overall visual differences between the groups and time points appear subtle, closer inspection indicates light-red regions concentrated in the center of the lyaseless and inactivated lyased monoculture groups (Groups 1 and 3) on day 10, suggesting localized cell death or reduced diffusion of nutrients in the scaffold core. This observation might reflect limitations in oxygen or nutrient penetration, particularly in dense or less-permeable scaffold structures.

The quantitative cell viability data in Figure 9 indicate that all the groups maintained high viability, with values consistently above 85% throughout the 10-day culture period. The CoreShell scaffold (Group 7) initially exhibited the lowest viability at approximately 85% on day 1. However, Group 7 showed a significant increase up to around 90% by day 10, suggesting that the dECM provides a more favorable environment for long-term cell growth and proliferation. Interestingly, the lyased coculture scaffold (Group 6) exhibited the highest and most consistent viability trends throughout the 10 days. This finding highlights that the combination of coculture and lyase treatment supplies optimal conditions for cellular health, likely due to enhanced nutrient and oxygen diffusion as well as synergistic interactions between cell types.

In conclusion, while all the groups maintained relatively high viability, the results emphasize the superior performance of the lyased scaffolds, particularly the coculture (Group 6) and CoreShell (Group 7) scaffolds. The gradual increase in viability of the Groups 6 and 7 further demonstrated the long-term benefits of this architecture, which supports sustained cellular activity and minimized cell death in the scaffold core. These findings collectively underline the critical role of scaffold architecture, treatment methods, and culture systems in promoting cell viability and enhancing the overall functionality of engineered tissues.

### 3.4. Toxicity and Biocompatibility of the Scaffolds

**Figure 10:**
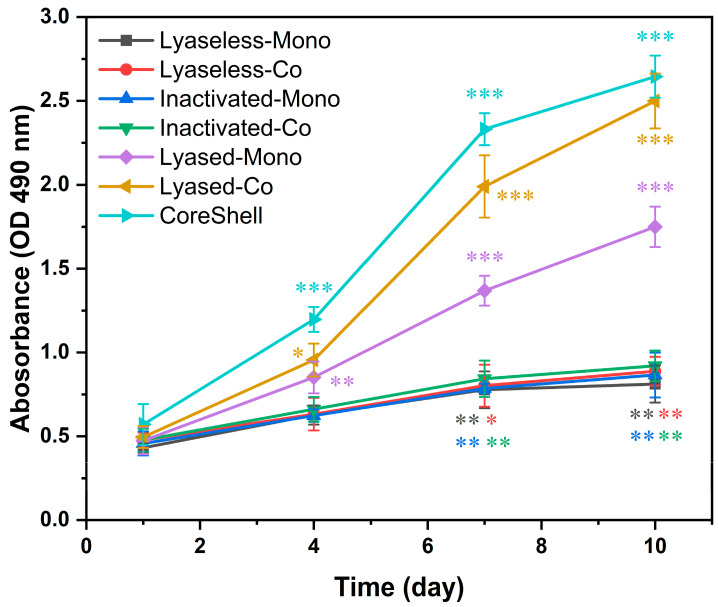
The toxicity and biocompatibility of the seven groups after 10 days of culture (* for *p* < 0.05, ** for *p* < 0.01, *** for *p* < 0.001, and *n* = 3).

The cytotoxicity of the engineered scaffolds was evaluated using the MTS assay to measure cell proliferation and metabolic activity across the seven groups on days 1, 4, 7, and 10, as shown in Figure 10. Absorbance values measured at 490 nm revealed distinct trends, reflecting the influence of scaffold composition, structural features, and cell configurations on cellular behavior.

Absorbance values increased over time in all the groups, indicating progressive cell proliferation and metabolic activity. Groups 1, 2, 3, and 4 exhibited similar trends, with gradual increases in absorbance values, suggesting consistent cell proliferation and good scaffold biocompatibility. The CoreShell scaffold (Group 7) demonstrated the highest metabolic activity, reaching an absorbance value of approximately 2.65 optical density (OD) on day 10. Similarly, the Lyased-Co group (Group 6) achieved the second-highest absorbance value (around 2.5 OD) by day 10, highlighting its enhanced ability to support cell viability and growth. In contrast, the lyaseless and inactivated groups (Groups 1, 2, 3, and 4) showed minimal increases in absorbance, remaining below 1.0 OD at the final time point.

The removal of alginate played a critical role in improving scaffold performance. The lyased groups (Groups 5 and 6) exhibited significantly higher absorbance values compared to the lyaseless and inactivated groups. The Lyased-Co group outperformed both the Lyaseless-Co and Inactivated-Co groups, demonstrating more than a 2.5-fold increase in metabolic activity by day 10. This improvement suggests that alginate removal enhanced nutrient and oxygen diffusion within the scaffold, creating a more favorable microenvironment for cell growth.

The inclusion of endothelial cells in the coculture groups further amplified scaffold performance. The coculture groups consistently outperformed their monoculture counterparts, with significant differences observed from day 4 onward. Notably, the Lyased-Co group demonstrated a superior performance compared to the Lyased-Mono group, achieving an absorbance value that was approximately 40% higher by day 10. These findings underscore the importance of vascularization in promoting metabolic activity and long-term cell viability.

Time-dependent trends highlighted the most significant differences between groups after day 4. By day 7, the CoreShell scaffold and Lyased-Co scaffold exhibited steep increases in absorbance, reflecting their superior capacity to sustain cell growth over time. In contrast, the lyaseless and inactivated groups showed limited improvement beyond day 4, indicating a reduced ability to support long-term cell viability.

### 3.5. Measurement of Mechanical Properties

**Figure 11:**
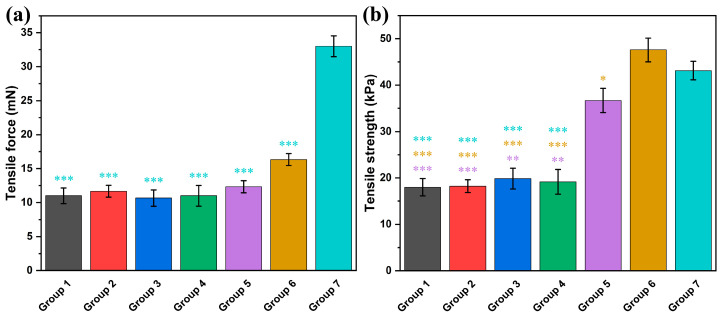
The mechanical properties (**a**) Tensile force, (**b**) Tensile strength of the seven groups after 10 days of culture (* for *p* < 0.05, ** for *p* < 0.01, *** for *p* < 0.001, and *n* = 3).

The tensile force and tensile strength of the seven scaffolds were measured to evaluate their mechanical properties. The tensile force values, recorded using a digital force gauge, are presented in Figure 11a, while the tensile strength, calculated by dividing the maximum tensile force by the cross-sectional area, is shown in Figure 11b.

For the lyaseless scaffolds (Groups 1 and 2), the tensile force remained relatively low, averaging around 11–12 mN, with corresponding tensile strengths of approximately 18 kPa. Similarly, the inactivated scaffolds (Groups 3 and 4) exhibited slightly improved tensile properties, with tensile strengths ranging between 19 and 20 kPa, despite having slightly lower tensile forces (around 11 mN).

Notably, the lyased scaffolds (Groups 5 and 6) demonstrated significant improvements in mechanical properties. Group 5 achieved a tensile force of approximately 12.3 mN and a tensile strength of 36.7 kPa, while Group 6 further increased to a tensile force of 16.3 mN and a tensile strength of 47.6 kPa. These results highlight the enhanced mechanical performance of the lyased scaffolds, likely due to the removal of alginate, which promoted tighter cell–hydrogel interactions and a more compact structure.

Group 7 (CoreShell) exhibited the highest tensile force, approximately 33 mN, but the second-highest tensile strength was around 43.1 kPa. This discrepancy is attributed to the larger diameter of its scaffold compared with the others, which reduced its tensile strength relative to its force.

### 3.6. Liver Function

**Figure 12:**
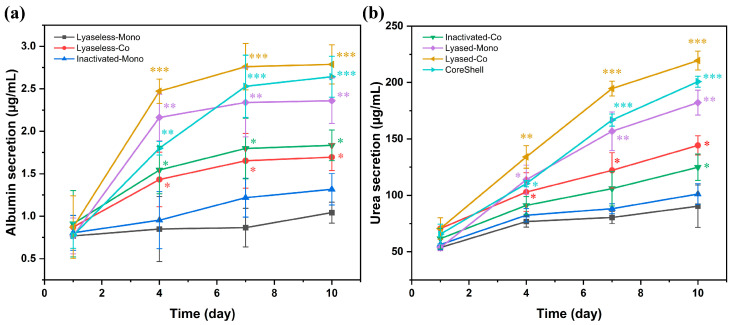
(**a**) The albumin secretion and (**b**) the urea secretion of all the seven scaffolds from day 1 to day 10 (* for *p* < 0.05, ** for *p* < 0.01, *** for *p* < 0.001, *n* = 3).

The albumin (Figure 12a) and urea secretion levels (Figure 12b) highlight significant trends in the metabolic performance of the seven scaffold groups over 10 days. Both albumin and urea secretion increased progressively across all the groups, but notable differences were observed in their rates and magnitudes. The Lyased-Co group (Group 6) consistently demonstrated the highest albumin and urea secretion throughout the experimental timeline, indicating its superior ability to support cell activity and metabolic output. Its albumin and urea secretion increased approximately 319.8% and 310.0%, respectively, from day 1 to day 10. This outcome can be attributed to the enhanced microenvironment provided by the lyased coculture system, which likely promoted cell–cell and cell–matrix interactions, thereby optimizing protein synthesis and metabolic processes.

The CoreShell group (Group 7) also showed substantial increases, particularly after day 4, suggesting a delayed but significant improvement in metabolic function. Its albumin and urea synthesis increased about 340.5% and 306.0%, respectively, from day 1 to day 10. Its larger diameter with higher cell numbers may have contributed to maintaining functionality over time, allowing for more sustained albumin and urea production.

In contrast, the Lyased-Mono group (Group 5) exhibited a marked improvement in both albumin and urea secretion (around 308.0% and 339.3%, respectively, from day 1 to day 10) compared to the two lyaseless groups (Groups 1 and 2). This suggests that the lyase treatment significantly enhanced scaffold porosity and nutrient diffusion, boosting cellular activity even in the monoculture condition. However, secretion levels in Group 5 were lower than those in Group 6 and 7, demonstrating that monoculture systems, even with the lyase treatment, are less effective than the coculture systems in promoting metabolic performance.

The inactivated lyased groups (Groups 3 and 4) showed an intermediate metabolic performance between the lyased and lyaseless scaffolds. Group 3 (Inactivated-Mono) demonstrated 26.4%- and 11.9%-higher albumin and urea secretion at day 10 compared to the Lyaseless-Mono group (Group 1), suggesting that even the inactivated alginate lyase treatment partially enhanced scaffold permeability and nutrient diffusion. However, the metabolic performance of Group 3 was significantly 28.2%- and 19.0%-lower at day 10 than that of Group 4, which used the coculture system.

The Inactivated-Co scaffolds (Group 4) exhibited improved metabolic outcomes compared to Group 3 and the lyaseless groups (Groups 1 and 2). Its coculture condition likely contributed to better cell–cell interactions and structural support, enhancing albumin and urea production. However, the secretion levels in Group 4 remained lower than those in the lyase-treated groups, highlighting the limited effect of the inactivated lyase treatment compared to the active lyase treatment in enhancing scaffold functionality.

The Lyaseless-Mono group (Group 1) consistently exhibited the lowest albumin and urea secretion throughout this study. This suggests that the absence of lyase treatment and the monoculture environment significantly limited cellular functionality, likely due to insufficient structural support or restricted cell growth.

In summary, the results demonstrate that scaffold structure, enzymatic treatment, and culture strategy significantly influence metabolic outcomes. The Lyased-Co group (Group 6) achieved the most optimal results, while the CoreShell group (Group 7) showed promising long-term performance improvements, particularly after day 4. These findings highlight the importance of combining scaffold design, enzymatic treatment, and coculture systems to enhance the metabolic functionality of engineered liver tissues.

### 3.7. Formation of Vascular Networks

**Figure 13:**
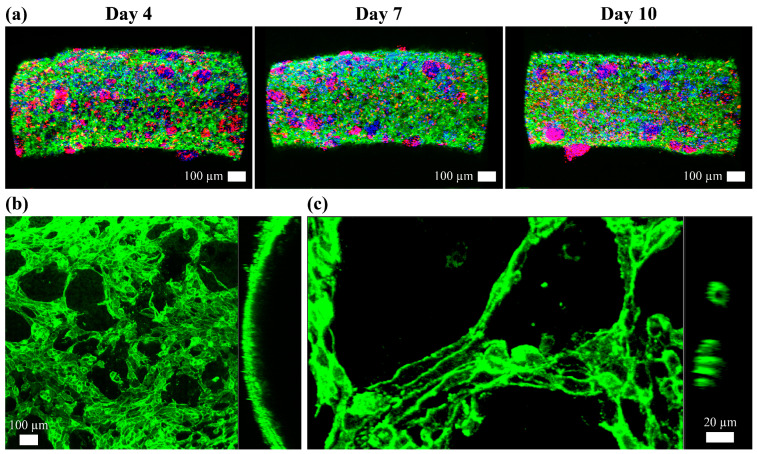
(**a**) The confocal images of the lyased coculture scaffold (Group 4) on day 4, 7, and 10. (Scale bar: 100 μm, red: albumin, green: CD-31, and blue: nuclei.) The higher-magnification images on day 10 with (**b**) the 50 μm scale bar and (**c**) the 20 μm scale bar.

The confocal images in Figure 13 demonstrated the progress of vascular network formation within the lyase coculture scaffold (Group 6) up to day 10. On day 4, CD-31 (the green in Figure 13a), a marker for endothelial cells, displayed a dense vascular network. On day 7, a more extensive and interconnected vascular network was observed. The increased intensity and distribution of the CD-31 fluorescence demonstrated endothelial cell proliferation and network formation. Concurrently, the red fluorescence for albumin was more pronounced, indicating enhanced hepatocyte function. The blue nuclei remained uniformly distributed, reflecting sustained cell viability. This suggests the scaffold effectively supports both endothelial and hepatic cells, promoting the formation of a functional vascular network. On day 10, the image demonstrated a well-established vascular network with dense and interconnected CD-31 fluorescence. The widespread albumin fluorescence signifies robust hepatocyte activity, and the uniformly distributed blue nuclei indicate ongoing cell viability and proliferation.

The higher magnification images in Figure 13b,c provided detailed views of the vascular structures inside the 10-day lyased coculture scaffold. Figure 13b highlighted the intricate and dense vascular network achieved with a lumen structure. Figure 13c emphasized the micro-vascular network formation. These results demonstrated that the coculture conditions and the application of lyase have successfully facilitated vascularization and hepatocyte functionality over time.

### 3.8. In Vivo Experiment

**Figure 14:**
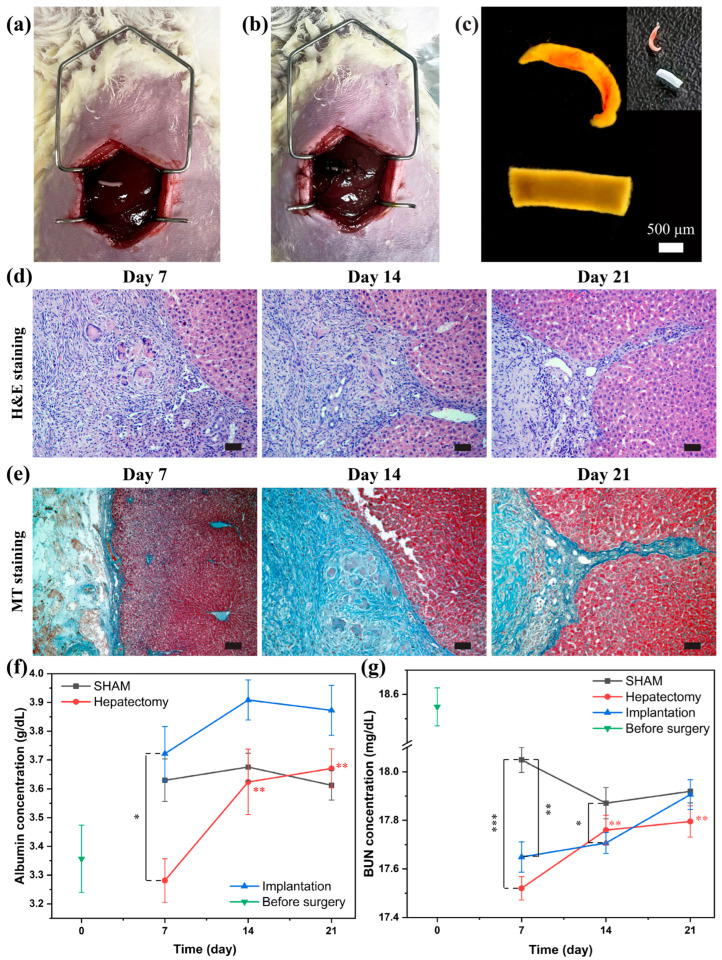
The animal implantation experiment. (**a**) The implantation position with the 10-day engineered scaffold (Group 6). (**b**) The engineered scaffold was inserted into the rat liver and sutured. (**c**) The image of the engineered scaffold and the excised rat liver. (**d**) The hematoxylin and eosin (H&E, pink: extracellular matrix, blue: nuclei) and (**e**) Masson’s trichrome (MT, blue: collagen fibers, red: cytoplasm, dark blue: nuclei) staining images of the retrieved and sliced tissue on day 7, 14, and 21 (scale bar: 50 μm). (**f**) The albumin concentration and (**g**) the blood urea nitrgen (BUN) concentration level of the implanted animal up to day 21 (* for *p* < 0.05, ** for *p* < 0.01, *** for *p* < 0.001, and *n* = 3).

Figure 14 exhibited surgical steps and analyzed results of the animal implantation experiment. After surgical removal of some portion of a liver, the 10-day lyased coculture scaffold (Group 6) was inserted and sutured in the extracted place, as shown in Figure 14a,b. Figure 14c provided visual representations of the engineered scaffold and the excised rat liver for comparison. The implanted scaffold had a diameter of 750 µm and a length of 3 mm, with an approximate volume of 1.33 mm^3^. The animal operator tried their best to cut out from a rat liver a similar volume to the engineered scaffold for the comparison before and after the implantation. Usually, four-week-old Sprague Dawley rats have a liver volume of approximately 9000 mm^3^ [31]. The excised liver volume might be approximately 1/6000 of the total volume of the host animal liver.

Through the 21-day observation period, the implanted rats had no swelling, no fever, and no infection. They have demonstrated continuous normal vitality and regular activity. On day 7, 14, and 21, blood samples and the implanted site were retrieved for quantitative and qualitative analysis. Figure 14d and e show the extracted tissue at the implanted site stained with hematoxylin and eosin (H&E) and Masson’s trichrome. Since day 7, a favorable connection and integration between the implanted scaffold and the host liver were shown. As time progressed up to day 21, the implanted scaffold demonstrated invasion into the host liver. Multiple perforations at every time point were observed in Figure 14d,e. These holes might mean ongoing blood vessel growth, which is crucial for the continued supply of nutrients and oxygen, thus preserving the artificial tissue’s viability within the living organism [32].

In Figure 14d, the blue things (Masson’s trichrome) became gradually denser from day 7 to 21. This indicated that collagen deposition increased as time progressed. Collagen is a major structural protein in the extracellular matrix (ECM), and it provides a platform for cells involved in wound healing, influencing their growth, survival, and movement [33,34,35]. The loose connective tissue like collagen exhibited a significant phenomenon in the implantation process [36,37]. This connective tissue plays an important role in maintaining tissue integrity, supporting organ function, and facilitating communication and transport within the body [38].

To evaluate the liver function of the implanted scaffold, albumin concentration, and blood urea nitrogen (BUN) levels in blood samples from the pre-surgery animal, the sham animal, the hepatectomy animal, and the implanted animal were quantified (Figure 14f,g). The normal range of albumin and BUN in Sprague Dawley rats is 2.9–4.8 g/dL and 13–29 mg/dL, respectively [39]. Prior to the implantation surgery, baseline albumin and BUN concentrations were measured as 3.357 ± 0.117 g/dL and 18.549 ± 0.078 mg/dL, respectively, confirming that the rats were in a healthy condition before the experimental procedures.

All the samples at every time point including the sample from the hepatectomy animal showed the normal range of albumin and BUN. As expected, the hepatectomy animal exhibited the lowest albumin and urea concentration on day 7, reflecting the impact of partial liver removal. However, the hepatectomy animal recovered its concentration level, similar to the sham animal, in a range of no significance at day 21. It is supposed that the excised liver volume ratio (1/6000) might be within the natural recovery capacity of the rat.

The implanted animal demonstrated a distinct trend. The albumin levels peaked on day 14, surpassing both the sham and hepatectomy groups, suggesting enhanced synthetic activity due to the supportive role of the implanted scaffold in liver function. By day 21, the albumin levels in the implanted group slightly decreased but remained higher than those in the sham group, indicating the sustained functional improvement. Similarly, BUN levels in the implanted group showed a gradual increase over time, reaching values comparable to the sham group by day 21, highlighting the scaffold’s positive impact on metabolic waste clearance. The data emphasize the potential of the implanted scaffold to enhance recovery and metabolic function in vivo.

## 4. Discussion

The confocal microscope image of Group 6 (the lyased coculture scaffold) demonstrated a tubular structure on day 10, as shown in Figure 13. It would be expected that the other coculture scaffolds (Groups 2, 4, and 7) have developed a vascular network with endothelial cells, even though they were not observed under confocal microscopy. In the diffusibility (the mean gray value), the albumin secretion, and the urea secretion, the coculture scaffolds (Groups 2, 4, 6, and 7) significantly surpassed the monoculture scaffolds (Groups 1, 3, and 5). The vascular network generated by the endothelial cells could make a more-secure pathway from the scaffold surface to the scaffold inside. This would enhance hepatic cell metabolism.

These vascular structures are stabilized by cell–matrix interactions, where integrins on the endothelial cell surface bind to extracellular matrix (ECM) components, such as fibronectin and collagen [40]. This interaction promotes cell adhesion, alignment, and sprouting, enabling the formation of functional vascular networks [41,42].

The cocultured endothelial cells influence hepatocyte metabolism through paracrine signaling by releasing growth factors, cytokines, and extracellular vesicles that regulate hepatic functions [43,44]. Key paracrine factors, such as vascular endothelial growth factor (VEGF) and hepatocyte growth factor (HGF), promote hepatocyte proliferation and enhance their metabolic activity [45,46]. Additionally, the direct interactions between endothelial cells and hepatocytes within the vascularized scaffold create a biomimetic microenvironment that sustains hepatocyte functionality over time [6].

Beyond paracrine signaling, cell–matrix interactions further support hepatocyte metabolism. The ECM provides biochemical and mechanical cues that activate intracellular signaling pathways, such as PI3K-Akt and ERK-MAPK, which are critical for cell survival and metabolic activity [47,48,49]. IntegrinECM interactions also regulate cytoskeletal organization, contributing to the maintenance of hepatocyte polarity and function [50].

Alginate, while beneficial in scaffold fabrication due to its fast gelation, could hinder molecule diffusion due to its dense structure [51]. In our research, the alginate component was dissolved at the lyased scaffolds (Groups 5, 6, and 7) 24 h after their extrusion. They showed a dramatic decrease in their diameter after the alginate removal. Without the alginate, the cells could bind better to the neighborhood hydrogel and cells, so that they could shrink by themselves. In the diffusibility (the mean gray value), the albumin secretion, and the urea secretion, the lyased scaffolds (Groups 5, 6, and 7) significantly surpassed the lyaseless scaffolds (Groups 1 and 2) and the inactivated scaffolds (Groups 3 and 4). The alginate-dissolving affected functionality of the liver scaffold much more than the coculturing of the endothelial cells.

The removal of alginate facilitated cell–matrix interactions by exposing cells to the underlying hydrogel matrix, which mimics the natural extracellular matrix (ECM). This allowed cells to form stronger adhesion sites and to promote migration, proliferation, and metabolic activity [34]. Specifically, the exposed ECM components, such as laminin and collagen, bind to integrin receptors on the cell surface, resulting in the formation of focal adhesions [52,53]. These structures serve as hubs for mechano-transduction, where mechanical signals from the ECM are converted into biochemical responses, enhancing cell proliferation and differentiation [54].

Right after the extrusion, the diameter of the CoreShell scaffold (Group 7) was over 2000 µm because of its outer-shell layer (Figure 5). This shell structure could hinder the cell medium’s diffusion into the cells encapsulated in the scaffold core. This might make its cell viability the lowest (approximately 85%) of all the seven groups on day 1. Due to the alginate removal 24 h after its extrusion, its viability increased gradually up to about 90% on day 10.

The core comprised the liver dECM alone with the hepatic cells and endothelial cells. Due to its abundant favorable environment provided by the liver dECM alone, Group 7 was one of the promising candidates in the liver functionality. However, the lyased coculture scaffold (Group 6) demonstrated a slightly better performance in the diffusibility and the urea secretion than Group 7. In the case of the albumin secretion, there is no significant difference between Groups 6 and 7. The space created by the alginate dissolving near the cell could enhance more cell proliferation, resulting in the better liver performance of Group 6. This slight difference was the reason why Group 6 was implanted.

The enhanced performance of Group 6 is attributed to improved cell–matrix interactions. The dissolving of alginate enabled cells to integrate more effectively with the surrounding ECM, facilitating the formation of an interconnected cellular network. This network replicates the native liver microenvironment, thereby optimizing cell functionality.

According to previous reports, an improved porosity and permeability in a scaffold matrix favorably impacted nutrient transport [3,4,11,55]. The cocultured endothelial cells have made vascular structures inside a scaffold, eventually aiding the diffusion [56,57]. Additionally, incorporating the liver dECM provided a biomimetic environment, promoting cellular attachment and migration, further contributing to efficient nutrient and waste exchange [58,59]. Our results align well with previous research.

No significant deviations in the albumin and urea secretion were observed within the implantation animal at any of the time points (Figure 14f,g). These findings indicate the successful implantation of the engineered tissue within the host rat liver. Over the 21-day observation period, the rats displayed continued vitality and regular activity. The histological staining (Figure 14d,e) showed an increase in collagen fibers with no separation between the engineered tissue and native liver tissue. Additionally, the artificial tissue has invaded the native tissue. It is supposed that the artificial and native tissue exhibited harmonious growth and integration. Importantly, no adverse symptoms were identified throughout the observation period, affirming the safety of our approach over three weeks. Furthermore, from day 7 the liver function of the implanted rats exhibited positive outcomes, demonstrating the effectiveness of our approach for long-term liver tissue engineering applications.

Despite the promising results, our model has two significant limitations to be addressed. Firstly, the model uses HepG2 cells, which do not fully replicate the functionality and behavior of primary human hepatocytes. This hinders the applicability of our engineered scaffold to replace diseased human livers. Secondly, the current structure is relatively simple and small. The simplicity of our scaffold design may not accurately mimic the complex architecture of the human liver, which is crucial for its proper function. Moreover, the small size of the evaluated scaffolds restricts its potential for use in therapeutic applications. To address these limitations, future work should focus on incorporating primary human hepatocytes and human hepatic sinusoidal endothelial cells in the fabrication process. Furthermore, efforts should be made to scale up for larger volumes, which is essential for creating clinically relevant liver replicates.

## 5. Conclusions

This study presents an approach to construct sub-millimeter-diameter artificial liver tissue by extruding the liver dECM and alginate mixture with endothelial cells and liver hepatocyte cells. The critical aspect of our strategy involved removing the alginate after scaffold formation, thereby directly influencing cellular behavior within the liver-dECM-alone microenvironment. Our findings demonstrated accelerated diffusion rates, enhanced albumin secretion and urea synthesis, and dense vascular network formation. The implantation of these scaffolds into animal models exhibited promising results for the development of functional liver tissue replacements.

## Figures and Tables

**Table 1:** The conditions and properties of the seven groups.

Group	Name	Structure	Extruding Material	Lyase Treatment	Cells
1	Lyaseless-Mono	Monolithic layer	Liver dECM—alginate (70:30)	No lyase	HepG2
2	Lyaseless-Co				HepG2—EA.hy926 (1:1)
3	Inactivated-Mono			Inactivated lyase-treated (0.05 units/mL)	HepG2
4	Inactivated-Co				HepG2—EA.hy926 (1:1)
5	Lyased-Mono			Lyase-treated (0.05 units/mL)	HepG2
6	Lyased-Co				HepG2—EA.hy926 (1:1)
7	CoreShell	Core–shell layer	Shell: alginate Core: Liver dECM		HepG2—EA.hy926 (1:1)

## Data Availability

The original contributions presented in the study are included in the article, further inquiries can be directed to the corresponding authors.

## References

[1] Han F., Wang J., Ding L., Hu Y., Li W., Yuan Z., Guo Q., Zhu C., Yu L., Wang H. (2020). Tissue Engineering and Regenerative Medicine: Achievements, Future, and Sustainability in Asia. Front. Bioeng. Biotechnol..

[2] Novosel E.C., Kleinhans C., Kluger P.J. (2011). Vascularization is the key challenge in tissue engineering. Adv. Drug Deliv. Rev..

[3] Rademakers T., Horvath J.M., van Blitterswijk C.A., LaPointe V.L.S. (2019). Oxygen and nutrient delivery in tissue engineering: Approaches to graft vascularization. J. Tissue Eng. Regen. Med..

[4] Rouwkema J., Koopman B., Blitterswijk C., Dhert W., Malda J. (2010). Supply of nutrients to cells in engineered tissues. Biotechnol. Genet. Eng. Rev..

[5] Lorente S., Hautefeuille M., Sanchez-Cedillo A. (2020). The liver, a functionalized vascular structure. Sci. Rep..

[6] Lv W., Zhou H., Aazmi A., Yu M., Xu X., Yang H., Huang Y.Y.S., Ma L. (2022). Constructing biomimetic liver models through biomaterials and vasculature engineering. Regen. Biomater..

[7] Hora S., Wuestefeld T. (2023). Liver Injury and Regeneration: Current Understanding, New Approaches, and Future Perspectives. Cells.

[8] Kryou C., Leva V., Chatzipetrou M., Zergioti I. (2019). Bioprinting for Liver Transplantation. Bioengineering.

[9] Mitchell G.M., Morrison W.A., Holnthoner W., Banfi A., Kirkpatrick J., Redl H. (2017). In Vitro and In Vivo Approaches for Pre-vascularization of 3-Dimensional Engineered Tissues. Vascularization for Tissue Engineering and Regenerative Medicine.

[10] Shokrani H., Shokrani A., Sajadi S.M., Seidi F., Mashhadzadeh A.H., Rabiee N., Saeb M.R., Aminabhavi T., Webster T.J. (2022). Cell-Seeded Biomaterial Scaffolds: The Urgent Need for Unanswered Accelerated Angiogenesis. Int. J. Nanomed..

[11] Sahoo D.R., Biswal T. (2021). Alginate and its application to tissue engineering. SN Appl. Sci..

[12] Rowley J.A., Madlambayan G., Mooney D.J. (1999). Alginate hydrogels as synthetic extracellular matrix materials. Biomaterials.

[13] Neves M.I., Moroni L., Barrias C.C. (2020). Modulating Alginate Hydrogels for Improved Biological Performance as Cellular 3D Microenvironments. Front. Bioeng. Biotechnol..

[14] Ali M., Payne S.L. (2021). Biomaterial-based cell delivery strategies to promote liver regeneration. Biomater. Res..

[15] Chu T.L., Tripathi G., Park M., Bae S.-H., Lee B.-T. (2022). In-vitro and in-vivo biocompatibility of dECM-alginate as a promising candidate in cell delivery for kidney regeneration. Int. J. Biol. Macromol..

[16] Lee J., Hong J., Kim W., Kim G.H. (2020). Bone-derived dECM/alginate bioink for fabricating a 3D cell-laden mesh structure for bone tissue engineering. Carbohydr. Polym..

[17] Huang C.-C. (2021). Characteristics and Preparation of Designed Alginate-Based Composite Scaffold Membranes with Decellularized Fibrous Micro-Scaffold Structures from Porcine Skin. Polymers.

[18] Wang J., Huang D., Yu H., Cheng Y., Ren H., Zhao Y. (2022). Developing tissue engineering strategies for liver regeneration. Eng. Regen..

[19] Tutty M.A., Movia D., Prina-Mello A. (2022). Three-dimensional (3D) liver cell models—A tool for bridging the gap between animal studies and clinical trials when screening liver accumulation and toxicity of nanobiomaterials. Drug Deliv. Transl. Res..

[20] Nguyen C.T., Duong V.T., Hwang C.H., Koo K.I. (2022). Angiogenesis in Free-Standing Two-Vasculature-Embedded Scaffold Extruded by Two-Core Laminar Flow Device. Int. J. Bioprint.

[21] Duong V.T., Nguyen C.T., Phan H.L., Le V.P., Dang T.T., Choi C., Seo J., Cha C., Back S.H., Koo K.-i. (2023). Double-layered blood vessels over 3 mm in diameter extruded by the inverse-gravity technique. Biofabrication.

[22] Duong V.T., Dang T.T., Kim J.P., Kim K., Ko H., Hwang C.H., Koo K.I. (2019). Twelve-day medium pumping into tubular cell-laden scaffold using a lab-made PDMS connector. Eur. Cell Mater..

[23] Duong V.T., Dang T.T., Hwang C.H., Back S.H., Koo K.I. (2020). Coaxial printing of double-layered and free-standing blood vessel analogues without ultraviolet illumination for high-volume vascularised tissue. Biofabrication.

[24] Koo K.I., Lenshof A., Huong L.T., Laurell T. (2020). Acoustic Cell Patterning in Hydrogel for Three-Dimensional Cell Network Formation. Micromachines.

[25] Le H.T., Phan H.L., Lenshof A., Duong V.T., Choi C., Cha C., Laurell T., Koo K.-i. (2024). Ultrasound standing wave spatial patterning of human umbilical vein endothelial cells for 3D micro-vascular networks formation. Biofabrication.

[26] Duong V.T., Dang T.T., Le V.P., Le T.H., Nguyen C.T., Phan H.L., Seo J., Lin C.-C., Back S.H., Koo K.-i. (2025). Direct extrusion of multifascicle prevascularized human skeletal muscle for volumetric muscle loss surgery. Biomaterials.

[27] Laschke M.W., Strohe A., Scheuer C., Eglin D., Verrier S., Alini M., Pohlemann T., Menger M.D. (2009). In vivo biocompatibility and vascularization of biodegradable porous polyurethane scaffolds for tissue engineering. Acta Biomater..

[28] Laschke M.W., Strohe A., Menger M.D., Alini M., Eglin D. (2010). In vitro and in vivo evaluation of a novel nanosize hydroxyapatite particles/poly(ester-urethane) composite scaffold for bone tissue engineering. Acta Biomater..

[29] Kurashina Y., Sato R., Onoe H. (2019). Microfiber-shaped building-block tissues with endothelial networks for constructing macroscopic tissue assembly. APL Bioeng..

[30] Alves D., Sileika T., Messersmith P.B., Pereira M.O. (2016). Polydopamine-Mediated Immobilization of Alginate Lyase to Prevent P. aeruginosa Adhesion. Macromol. Biosci..

[31] Noorafshan A., Esmail-Zadeh B., Bahmanpour S., Poost-Pasand A. (2005). Early stereological changes in liver of Sprague-Dawley rats after streptozotocin injection. Indian. J. Gastroenterol..

[32] Apelgren P., Amoroso M., Säljö K., Montelius M., Lindahl A., Stridh Orrhult L., Gatenholm P., Kölby L. (2021). Vascularization of tissue engineered cartilage—Sequential in vivo MRI display functional blood circulation. Biomaterials.

[33] Diller R.B., Tabor A.J. (2022). The Role of the Extracellular Matrix (ECM) in Wound Healing: A Review. Biomimetics.

[34] Wight T.N., Potter-Perigo S. (2011). The extracellular matrix: An active or passive player in fibrosis?. Am. J. Physiol. Gastrointest. Liver Physiol..

[35] Wang T., Fu X., Jin T., Zhang L., Liu B., Wu Y., Xu F., Wang X., Ye K., Zhang W. (2019). Aspirin targets P4HA2 through inhibiting NF-κB and LMCD1-AS1/let-7g to inhibit tumour growth and collagen deposition in hepatocellular carcinoma. eBioMedicine.

[36] Janeczek M., Szymczyk P., Dobrzynski M., Parulska O., Szymonowicz M., Kuropka P., Rybak Z., Zywicka B., Ziolkowski G., Marycz K. (2018). Influence of surface modifications of a nanostructured implant on osseointegration capacity—Preliminary in vivo study. RSC Adv..

[37] Massari K.V., Marinho G.O., Silva J.L., Holgado L.d.A., Leão A.L., Chaves M.R.M., Kinoshita A. (2015). Tissue reaction after subcutaneous implantation of a membrane composed of bacterial cellulose embedded with hydroxyapatite. Dent. Oral Craniofacial Res..

[38] Kamrani P., Marston G., Arbor T.C., Jan A. (2024). Anatomy, Connective Tissue. StatPearls.

[39] Loeb W.F., Quimby F. (1999). The Clinical Chemistry of Laboratory Animals.

[40] Winkler J., Abisoye-Ogunniyan A., Metcalf K.J., Werb Z. (2020). Concepts of extracellular matrix remodelling in tumour progression and metastasis. Nat. Commun..

[41] Mettouchi A. (2012). The role of extracellular matrix in vascular branching morphogenesis. Cell Adh Migr..

[42] Francis M.E., Uriel S., Brey E.M. (2008). Endothelial cell-matrix interactions in neovascularization. Tissue Eng. Part B Rev..

[43] Jindal R., Nahmias Y., Tilles A.W., Berthiaume F., Yarmush M.L. (2009). Amino acid-mediated heterotypic interaction governs performance of a hepatic tissue model. Faseb J..

[44] Sakaguchi T.F., Sadler K.C., Crosnier C., Stainier D.Y. (2008). Endothelial signals modulate hepatocyte apicobasal polarization in zebrafish. Curr. Biol..

[45] Jin Y., Guo Y.H., Li J.C., Li Q., Ye D., Zhang X.X., Li J.T. (2023). Vascular endothelial growth factor protein and gene delivery by novel nanomaterials for promoting liver regeneration after partial hepatectomy. World J. Gastroenterol..

[46] Fujimori H., Asahina K., Shimizu-Saito K., Ikeda R., Tanaka Y., Teramoto K., Morita I., Teraoka H. (2008). Vascular endothelial growth factor promotes proliferation and function of hepatocyte-like cells in embryoid bodies formed from mouse embryonic stem cells. J. Hepatol..

[47] Nakayama K.H., Hou L., Huang N.F. (2014). Role of extracellular matrix signaling cues in modulating cell fate commitment for cardiovascular tissue engineering. Adv. Health Mater..

[48] Pickup M.W., Mouw J.K., Weaver V.M. (2014). The extracellular matrix modulates the hallmarks of cancer. EMBO Rep..

[49] Zalpoor H., Aziziyan F., Liaghat M., Bakhtiyari M., Akbari A., Nabi-Afjadi M., Forghaniesfidvajani R., Rezaei N. (2022). The roles of metabolic profiles and intracellular signaling pathways of tumor microenvironment cells in angiogenesis of solid tumors. Cell Commun. Signal..

[50] Masuzaki R., Ray K.C., Roland J., Zent R., Lee Y.A., Karp S.J. (2021). Integrin β1 Establishes Liver Microstructure and Modulates Transforming Growth Factor β during Liver Development and Regeneration. Am. J. Pathol..

[51] Puguan J.M.C., Yu X., Kim H. (2015). Diffusion characteristics of different molecular weight solutes in Ca–alginate gel beads. Colloids Surf. A Physicochem. Eng. Asp..

[52] Kleiser S., Nyström A. (2020). Interplay between Cell-Surface Receptors and Extracellular Matrix in Skin. Biomolecules.

[53] Chastney M.R., Conway J.R.W., Ivaska J. (2021). Integrin adhesion complexes. Curr. Biol..

[54] Jansen K.A., Atherton P., Ballestrem C. (2017). Mechanotransduction at the cell-matrix interface. Semin. Cell Dev. Biol..

[55] Karande T.S., Ong J.L., Agrawal C.M. (2004). Diffusion in musculoskeletal tissue engineering scaffolds: Design issues related to porosity, permeability, architecture, and nutrient mixing. Ann. Biomed. Eng..

[56] Goers L., Freemont P., Polizzi K.M. (2014). Co-culture systems and technologies: Taking synthetic biology to the next level. J. R. Soc. Interface.

[57] Kuppusamy P., Kim D., Soundharrajan I., Hwang I., Choi K.C. (2020). Adipose and Muscle Cell Co-Culture System: A Novel In Vitro Tool to Mimic the In Vivo Cellular Environment. Biology.

[58] Lee H., Han W., Kim H., Ha D.-H., Jang J., Kim B.S., Cho D.-W. (2017). Development of Liver Decellularized Extracellular Matrix Bioink for Three-Dimensional Cell Printing-Based Liver Tissue Engineering. Biomacromolecules.

[59] Meng F., Assiri A., Dhar D., Broering D. (2017). Whole liver engineering: A promising approach to develop functional liver surrogates. Liver Int..

